# Improved Environment-Aware–Based Noise Reduction System for Cochlear Implant Users Based on a Knowledge Transfer Approach: Development and Usability Study

**DOI:** 10.2196/25460

**Published:** 2021-10-28

**Authors:** Lieber Po-Hung Li, Ji-Yan Han, Wei-Zhong Zheng, Ren-Jie Huang, Ying-Hui Lai

**Affiliations:** 1 Department of Otolaryngology Cheng Hsin General Hospital Taipei Taiwan; 2 Faculty of Medicine, Institute of Brain Science National Yang Ming Chiao Tung University Taipei Taiwan; 3 Department of Medical Research China Medical University Hospital China Medical University Taichung Taiwan; 4 Department of Speech Language Pathology and Audiology, College of Health Technology National Taipei University of Nursing and Health Sciences Taipei Taiwan; 5 Department of Biomedical Engineering National Yang Ming Chiao Tung University Taipei Taiwan

**Keywords:** cochlear implants, noise reduction, deep learning, noise classification, hearing, deaf, sound, audio, cochlear

## Abstract

**Background:**

Cochlear implant technology is a well-known approach to help deaf individuals hear speech again and can improve speech intelligibility in quiet conditions; however, it still has room for improvement in noisy conditions. More recently, it has been proven that deep learning–based noise reduction, such as noise classification and deep denoising autoencoder (NC+DDAE), can benefit the intelligibility performance of patients with cochlear implants compared to classical noise reduction algorithms.

**Objective:**

Following the successful implementation of the NC+DDAE model in our previous study, this study aimed to propose an advanced noise reduction system using knowledge transfer technology, called NC+DDAE_T; examine the proposed NC+DDAE_T noise reduction system using objective evaluations and subjective listening tests; and investigate which layer substitution of the knowledge transfer technology in the NC+DDAE_T noise reduction system provides the best outcome.

**Methods:**

The knowledge transfer technology was adopted to reduce the number of parameters of the NC+DDAE_T compared with the NC+DDAE. We investigated which layer should be substituted using short-time objective intelligibility and perceptual evaluation of speech quality scores as well as *t*-distributed stochastic neighbor embedding to visualize the features in each model layer. Moreover, we enrolled 10 cochlear implant users for listening tests to evaluate the benefits of the newly developed NC+DDAE_T.

**Results:**

The experimental results showed that substituting the middle layer (ie, the second layer in this study) of the noise-independent DDAE (NI-DDAE) model achieved the best performance gain regarding short-time objective intelligibility and perceptual evaluation of speech quality scores. Therefore, the parameters of layer 3 in the NI-DDAE were chosen to be replaced, thereby establishing the NC+DDAE_T. Both objective and listening test results showed that the proposed NC+DDAE_T noise reduction system achieved similar performances compared with the previous NC+DDAE in several noisy test conditions. However, the proposed NC+DDAE_T only required a quarter of the number of parameters compared to the NC+DDAE.

**Conclusions:**

This study demonstrated that knowledge transfer technology can help reduce the number of parameters in an NC+DDAE while keeping similar performance rates. This suggests that the proposed NC+DDAE_T model may reduce the implementation costs of this noise reduction system and provide more benefits for cochlear implant users.

## Introduction

Cochlear implants (CIs) are implanted electronic medical devices that can enable patients with profound-to-severe hearing loss to obtain a sense of sound. In their study, Gifford et al [1] showed that 28% of individuals equipped with CI achieved 100% speech intelligibility. Sladen et al [2] also reported similar results in their study: after undergoing CI implantation, the word accuracy of CI users was 80% in a quiet environment. Although CI users have few obstacles in a quiet environment, there is still scope for improvement in a noisy environment [2].

Noise reduction (NR) is one of classical methods to alleviate the effect of background noise for CI users. Over the past few decades, many statistical signal processing NR methods have been proposed, such as log minimum mean squared error [3], Karhunen-Loéve transform [4], Wiener filter-based on a priori signal-to-noise ratio (SNR) estimation [5], generalized maximum a posteriori spectral amplitude [6], and SNR-based [7] approaches. Loizou et al [8] proposed a single-channel algorithm to conduct NR, and the results showed that the sentence recognition scores in 14 participants with CI improved significantly over their daily performances. Dawson et al [7] evaluated a real-time NR algorithm which used the noise estimation to pick up 1 NR approach out of 2 different levels of NR approaches according to the SNR. The study results showed that the proposed NR algorithm could benefit CI users in speech a reception threshold under 3 kinds of noise. Mauger et al [9] optimized the gain function to achieve a better SNR-based NR, and the results showed that with the optimized gain function, a 27% improvement was achieved for CI users in speech-weighted noise. Although classical NR function can improve speech intelligibility for CI users in stationary noise conditions [7-9], improvements are still needed in nonstationary noise conditions [10].

Deep learning (DL)–based NR methods have recently shown better performance than classical statistical-based NR methods [11-17]. Lai et al [18] used a deep denoising autoencoder (DDAE)–based NR using vocoder simulation to perform NR function for CI users; the listening test showed that the speech intelligibility was better with DDAE-based NR than with convectional single-microphone NR approaches, whether in stationary or nonstationary noise conditions. Goehring et al [19,20] used neural and recurrent neural networks to perform the NR function for CI users, and the results showed that the proposed NR function could significantly improve speech intelligibility in babbling noise conditions. In DL methods, the nonstationary noise can be processed well, but this needs a huge amount of training data in different noise types and SNR levels. However, when a mismatch exists, such as when there is a difference in data between the training and testing phase, the performance of the DL method is usually degraded [10,18].

An environment-aware–based NR system called noise classifier (NC) +DDAE (NC+DDAE) was proposed to alleviate the above issue [21]. The NC+DDAE NR system combines *n*-specific noise-dependent (ND)-DDAE NR models and a noise-independent (NI)-DDAE NR model. The NC function (ie, deep neural network model) was used to distinguish *n* different typical noises and select a suitable DDAE model to perform the NR function for CI users. Hence, the NC function made the NC+DDAE an environment-aware–based NR system. The objective measures and listening test showed that the NC+DDAE model had a much higher performance than did the other NR methods. Although the NC+DDAE model has proven to benefit the CI user and have the flexibility of customization, the NC+DDAE model requires several parameters, which increase the requirements for device implementation. Therefore, the NC+DDAE model needs to be modified to have fewer requirements while maintaining the performance at the same level.

Recently, the knowledge transfer (so called transfer learning) approach [22] has been used in many speech signal processing tasks (eg, speech emotion detection [23], text-to-speech system [24,25], and speech enhancement [26]) and has proven to provide benefits for the DL-based model. Knowledge transfer is a machine learning method developed for a specific task that reuses the initial parameters for a new model for the target task. In other words, the knowledge transfer technology transfers the domain knowledge based on the source domain to the target domain to help the DL-based model achieve better performance; furthermore, it can speed up the time needed to develop and train a model by reusing these pieces or modules that have already been developed [22]. Following the concept of knowledge transfer technology, we proposed an improved NC+DDAE NR model, called NC+DDAE_transfer (NC+DDAE_T). We first analyzed the differences between features in each layer of DDAE to choose the most suitable layer for NR adaptation. Next, we compared the performance between NC+DDAE and NC+DDAE_T with 2 well-known objective metrics: perceptual evaluation of speech quality (PESQ) [27] and short-time objective intelligibility (STOI) [28]. The PESQ shows the result of comparing the clean and processed speech by mean opinion score. In the mean opinion score, 5 is the highest score while 1 is the lowest. According to a previous study [27], a score over 4 is high enough for most people to listen comfortably and a score of 3.6 is an acceptable boundary for those with normal hearing. The STOI represents the speech intelligibility by a correlation coefficient derived from comparing the energy of clean and processed speech in each frame. STOI ranges from 0 to 1, with a higher score representing more clear and understandable speech. Finally, the clinical effectiveness of NC+DDAE_T with the NC+DDAE and DDAE NR systems for patients with CI was evaluated in noisy listening conditions.

## Methods

In this section, we describe first the NC+DDAE approach. We then introduce the NC+DDAE_T method, the transfer learning–based NC+DDAE NR modified in this study. Finally, we describe the experimental setting and material to prove the benefits of the proposed NC+DDAE_T compared to 2 well-known DL-based NR systems (ie, DDAE and NC+DDAE).

### NR Based on the NC+DDAE Approach

**Figure 1:**
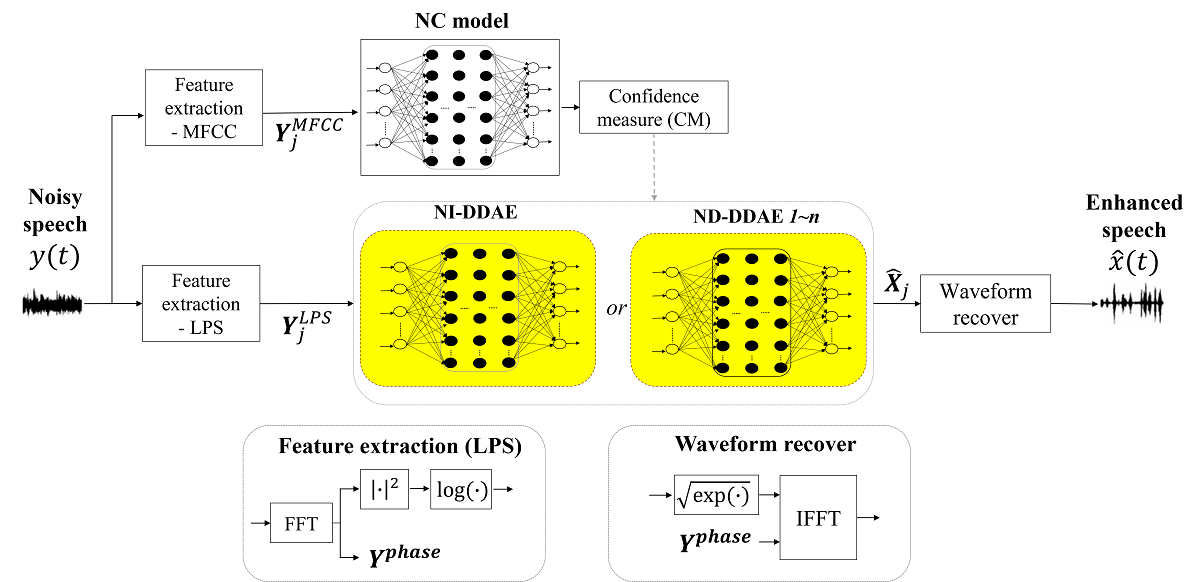
Structure of the noise classifier with a deep denoising autoencoder (NC+DDAE) system. DDAE: deep denoising autoencoder; FFT: fast Fourier transform; IFFT: inverse fast Fourier transform; LPS: log power spectra; NC: noise classifier; ND: noise-dependent; NI: noise-independent; MFCC: Mel-frequency cepstral coefficient.

Figure 1 shows the proposed NC+DDAE model in our previous study [21], where 2 critical units, NC and DDAE, were included. In this approach, first, the noisy speech signals ***y(t)*** are processed by feature extraction units to obtain ***Y_j_^MFCC^*** and ***Y_j_^LPS^***, which denote log power spectra (LPS) [29] and Mel-frequency cepstral coefficients [30], respectively, with *j* denoting the frame in the short-time Fourier transform. ***Y_j_^MFCC^*** is the input of the NC model to determine the current type of background noise and to select a suitable DDAE model for NR, which includes multiple ND-DDAE models each trained by a model-specific noise type and a single NI-DDAE model trained by 120 noise types [15]. When the noisy input signal is similar to one of the specific noise types, the specific ND-DDAE model is chosen for NR; otherwise, the NI-DDAE is used. Afterward, the selected DDAE model processes ***Y_j_^LPS^*** to obtain the enhanced features. 
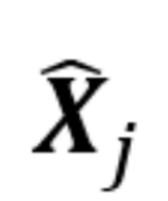
 is combined with the noisy phase ***Y^phase^*** to finally reconstruct the enhanced speech 
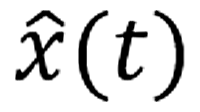
. The NC+DDAE NR system has been defined in detail previously [21].

### NR With the Proposed NC+DDAE_T Approach

**Figure 2:**
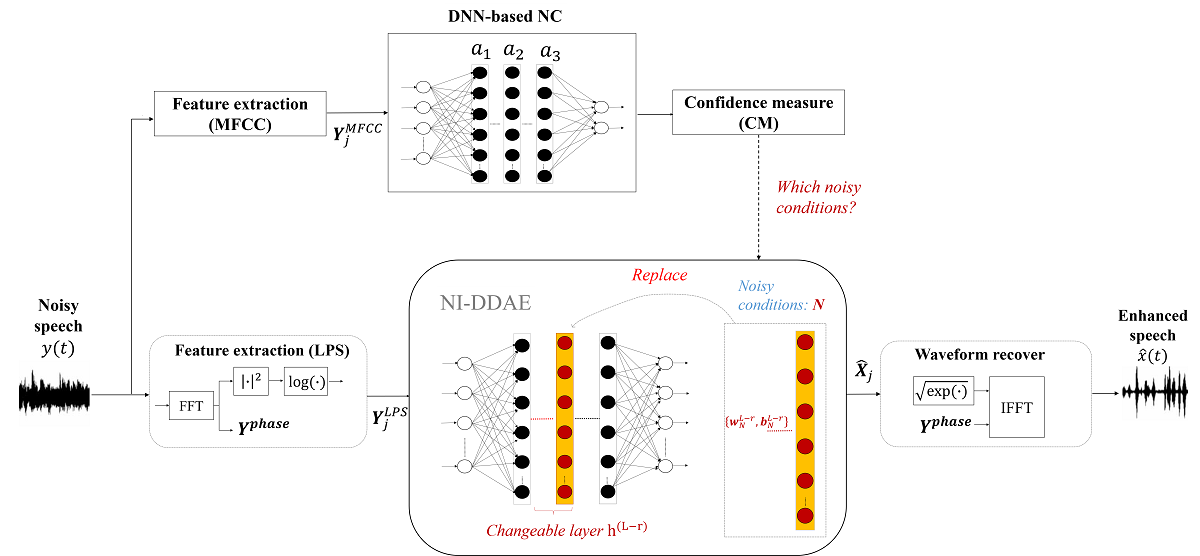
Structure of the proposed noise classifier system with DDAE and knowledge transfer. DDAE: deep denoising autoencoder; DNN: deep neural network; FFT: fast Fourier transform; IFFT: inverse fast Fourier transform; LPS: log power spectra; NC: noise classifier; NI: noise-independent; MFCC: Mel-frequency cepstral coefficient.

Figure 2 shows the pipeline of the NC+DDAE_T NR approach proposed in this study. The signal processing procedure of the NC+DDAE_T is similar to that of the above-mentioned NC+DDAE. The major difference lies in the NR model as described in the following sections.

#### NC Model

The NC model of the proposed NC+DDAE_T is the same as that in our previously described system. Initially, the system receives a noisy speech ***y(t)*** and computes the ***Y_j_^MFCC^*** and ***Y_j_^LPS^*** features separately. ***Y_j_^MFCC^*** is then sent to the NC model. The NC model is a deep neural network (DNN) composed of 3 hidden layers. Each layer consists of 100 neurons and an output layer adapting the softmax function [30]. The output at the *j-*th node of the *l-*th layer in a DNN h*_j_*^(^*^l^*^)^ is produced according to equation 1:


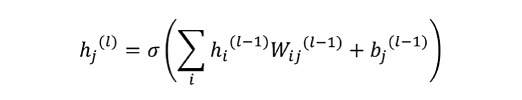
 (**1**)

where the term *h_j_^(l–1)^* denotes the output from the *i*-th node in the (*l*−1)-th layer, *b_j_^(l)^* is the bias of index *j*, and *W_ij_^l^* is the weight between hidden unit *j* and *i*. σ*(∙)* is the activation function [30], which is the logistic function described in equation 2:


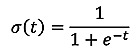
 (**2**)

Next, the trained DNN model is used in the NC function. The output of the last layer is converted into the probability by the softmax function [31] to obtain the normalized probability-based output. The back propagation algorithm [32,33] is then applied to parameter set θ in equation 3, where *L*(∙) is the loss function, *N_i_* denotes the correct noise class, and 
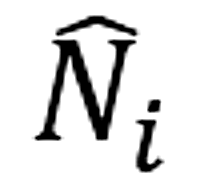
 is the output class of the DNN-based NC.


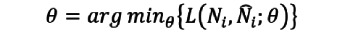
 (**3**)

To avoid substantial variance in the DNN output, we use the confidence measurement [34] to analyze the output of the DNN-based NC. Based on the confidence measurement score, a threshold is used to determine the classification results. In other words, when the confidence measurement score is higher than the threshold, the result predicted by the NC model is considered trustworthy. Nevertheless, if the confidence measurement score is not concrete to one noise type, then the NI-DDAE is chosen for NR; on the other hand, if the confidence measurement is solid, the ND-DDAE is selected.

#### DDAE-based NR Model

In the training phase, the noisy LPS feature ***Y_j_^LPS^*** and clean LPS feature ***X_j_^LPS^*** are the input and output, respectively, of the DDAE–based NR model. The details for training the DDAE NR model with *L* hidden layers mapping ***Y_j_^LPS^*** to ***X_j_^LPS^*** are available elsewhere [21]. The difference between NC+DDAE and NC+DDAE_T is that only the parameters of a specific layer (ie, *w^L-r^* and *b^L-r^*) are trainable as shown in equation 4, whereas the other parameters remain untrainable in the fine-tuning process. The constant *L* denotes the number of layers, and we used 5 layers (ie, *L*=5) in this study.


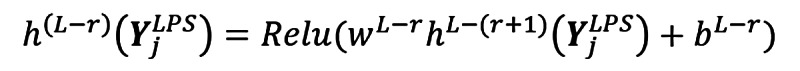
 (**4**)

where {*W*^1^…*W*^(L-r)^…*W^L^*} and {*b*^1^… *b*^(^*^L^*^-r)^… *b^L^*} are the matrices of weights and bias vectors of the DDAE NR model, respectively, whereas *Relu* represents the activation function rectified linear unit [35]. The constant *r* is the index to identified the specific trainable layer. In this study, the second layer (ie, *r*=3) was chosen because, on average, substituting the second layer achieved the best performance in our pilot study. The detailed experimental results are shown in Multimedia Appendix 1.

Based on the above idea, the original NI-DDAE, trained with a huge database of noise samples, can be transformed into many ND-DDAE models according to the type of background noise. In this study, 12 common types of background noise were used; hence, 12 ND-DDAE models were derived from the NI-DDAE model. More specifically, each ND-DDAE model was determined by optimizing the following objective function:


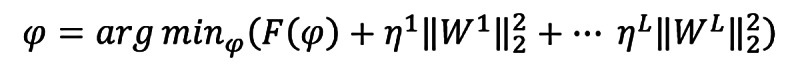
 (**5**)


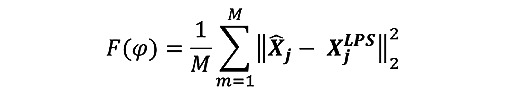
 (**6**)

where *M* is the total number of training samples and F(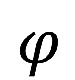) is the loss function derived from 
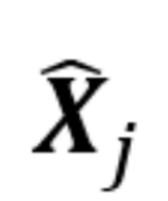
 and ***X_j_^LPS^_._*** 
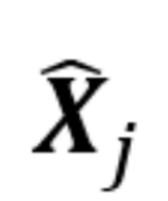
 is the vector that contains the logarithmic amplitudes of the enhanced speech corresponding to the paired noisy LPS feature ***Y_j_^LPS^***. Subsequently, the trained NI-DDAE provides the initial parameters for the ND-DDAE model, and the noise data of the specific environment are used to fine-tune this ND-DDAE model. Finally, the transformed LPS feature 
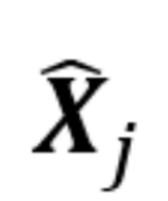
 is sent to the waveform recovery unit to reconstruct the waveform. More specifically, 
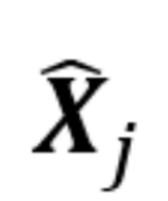
 is first processed using square root and exponential operations. The waveform recovery function then reconstructs the enhanced speech 
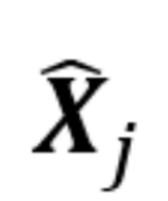
 with the noisy phase ***Y^phase^***.

### Training and Evaluation Procedure

**Figure 3:**
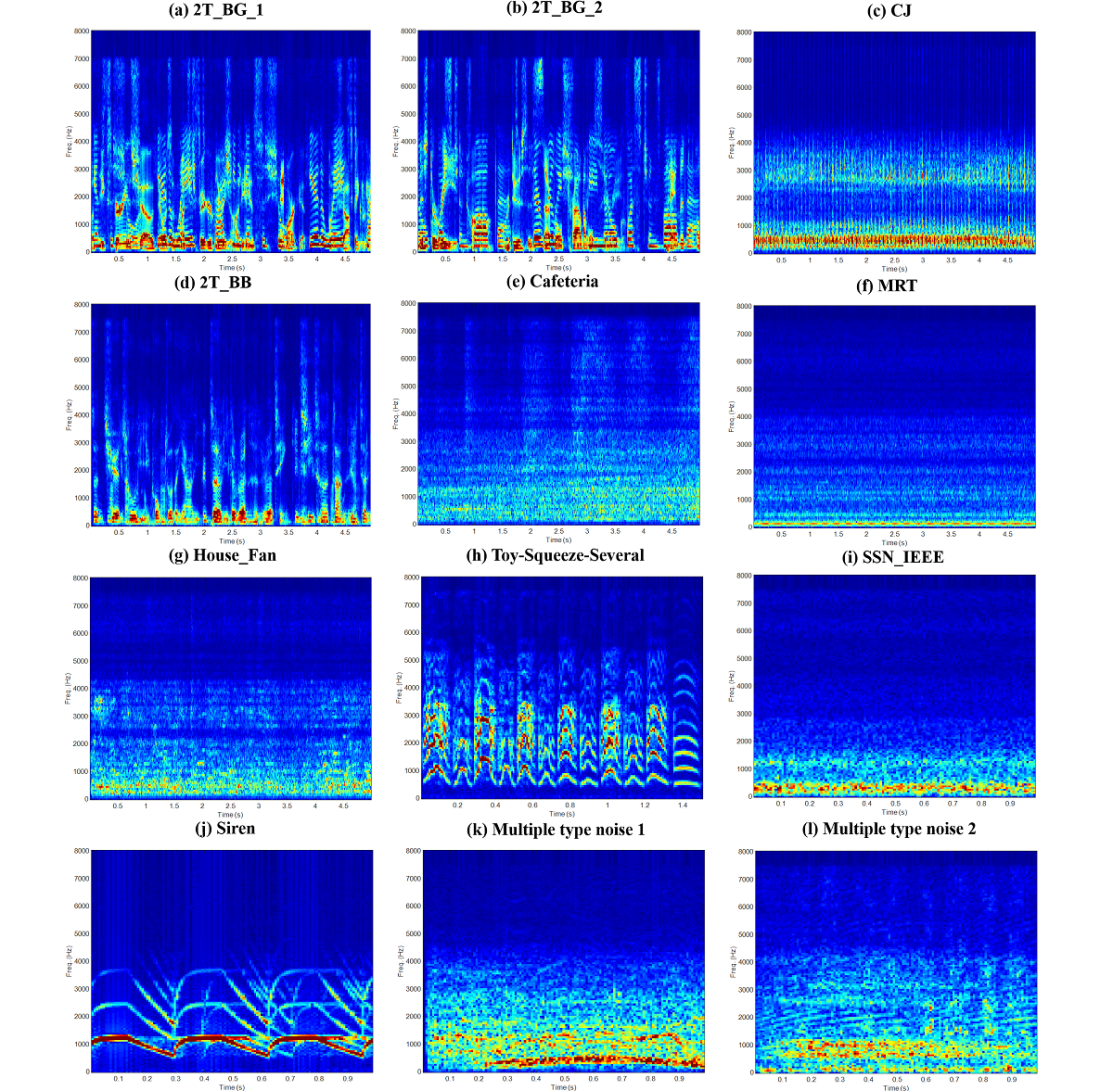
Spectrograms of the 12 noise signals: (a) 2T_BG_1, (b) 2T_BG_2, (c) CJ, (d) 2T_BB, (e) Cafeteria, (f) MRT, (g) House Fan, (h) Toy-Squeeze-Several, (i) SSN_IEEE, (j) Siren, (k) Multiple type noise 1, and (l) Multiple type noise 2. 2T_BG_1 is a noise that mixes the speech of a girl and a boy both speaking repeatedly in English. 2T_BG_1 is a noise that mixes the speech of a girl and a boy both speaking repeatedly in English. The speakers in 2T_BG_2 are the same as those in 2T_BG_1 but with different sentences. 2T_BB is a noise that overlays 2 sentences in Chinese spoken by the same male speaker. Multiple type noise 1 is a mix of the sound of sirens and cheering crowd, whereas Multiple type noise 2 is a sound combining scratching and booing. The other samples are common background noises from daily life. 2T_BB: 2 Talker; 2T_BG_1: 2 talker_unseen1; 2T_BG_2: 2 talker_unseen2; CJ: Construction Jackhammer; MRT: Mass Rapid Transit; SSN_IEEE: speech shape noise from the Institute of Electrical and Electronics Engineers.

In this section, we show how the NC, DDAE, and NC+DDAE_T models were trained. First, we trained a new NC model according to the 12 common background noises, 2talker_unseen1, 2talker_unseen2, Construction Jackhammer (CJ), 2 Talker, Cafeteria, MRT (Mass Rapid Transit), cafeteria, Toy-Squeeze-Several, speech shape noise from the Institute of Electrical and Electronics Engineers (SSN_IEEE), Siren, Multiple type noise 1, and Multiple type noise 2, which are shown in Figure 3. Note that the training approach is described in the previous section “NC Model”. After the training, the prediction accuracy of the 12 noises was 100%. The detailed results of the confusion matrix are shown in Multimedia Appendix 2.

To train the DDAE NR model, the Taiwan Mandarin version of the hearing in noise test (TMHINT) corpus [36] was selected to conduct all experiments, including the training and evaluation parts. All 320 sentences, each consisting of 10 characters, were recorded at a 16 kHz sampling rate, after which 120 utterances among the TMHINT corpus were selected and corrupted by 120 noise types [15] at 7 SNR levels (−10, −7, −4, −1, 1, 4, 7, and 10 dB) as the training set for the DDAE model. The other 200 utterances were also corrupted with the 12 common background noises—as mentioned in the description of NC training—at 6 SNR levels (-6, -3, 0, 3, and 6 dB) as the outside testing set. In our previous study, this trained model was defined as the NI-DDAE.

Next, we combined the NC with NI-DDAE and fine-tuned the model with each noise type in the NC, and the NI-DDAE was transformed into NC+DDAE_T. In the fine-tuning step, we could freeze or adopt each layer in the NI-DDAE. Previously, we had studied which layer of the NI-DDAE model had to be replaced to achieve the best performance. We substituted each layer by modifying *r* in the range from 1 to 5; meanwhile, we conducted 2 well-known objective speech evaluations, PESQ [27] and STOI [28], to identify the most appropriate layer. On average, replacing the middle layer of the NI-DDAE model (ie, the second layer this study) achieved a better performance than did substituting other layers. The detailed results can be found in Multimedia Appendix 1. Hence, we uniformly replaced the parameters of the third layer in all subsequent tests. As the 2 DL-based NR systems, DDAE and NC+DDAE, achieved better performances in our previous studies [18,21] than did the well-known unsupervised NR algorithms, the log minimum mean squared error [3] and Karhunen-Loéve transform [37], we used the DDAE and NC+DDAE algorithms for comparisons to evaluate the NC+DDAE_T in this study.

Subsequently, we enrolled 10 CI users to conduct speech intelligibility tests, and details of these subjects are shown in the Multimedia Appendix 3. This study protocol was approved by the Research Ethics Review Committee of Cheng Hsin Hospital under the following approval number: CHGH-IRB (645) 107A-17-2. The first author, LPHL, explained the study to the patients and collected the signed institutional review board informed consent before the experiment. All participants used their own clinical speech processors and temporarily disabled the built-in NR functions during the test. The test signals of noisy and enhanced speech were played at 65 dB sound pressure level by a speaker and were then processed through a CI processor to simulate the performance of each NR approach for CI users. To ensure that fatigue did not affect the study participants, each individual only heard a total of 16 test conditions (2 background noise [2 talker and CJ] × 2 SNR levels [0 and 3 dB] × 4 signal processing systems [noisy, DDAE, NC+DDAE, and NC+DDAE_T]) with 10 sentences of 10 words in each test condition. The participants were instructed to repeat verbally what they had heard. We evaluated the speech intelligibility under each test condition using the word correct rate (WCR) [38-42] calculated as the ratio between the number of correctly identified words and the total number of words. To further prevent participant fatigue, tests were paused for 5 minutes every 30 minutes. Moreover, we calculated the statistical power to see whether the sample size (10 patients in this study) was large enough to obtain a significant difference in the result. The statistical power of this study is 1. According to Cohen et al [43] a statistical power over 0.8 is sufficiently high to conclude that there is a significant difference in the hypothesis.

## Results

### Objective Evaluation Using PESQ and STOI Scores

**Figure 4:**
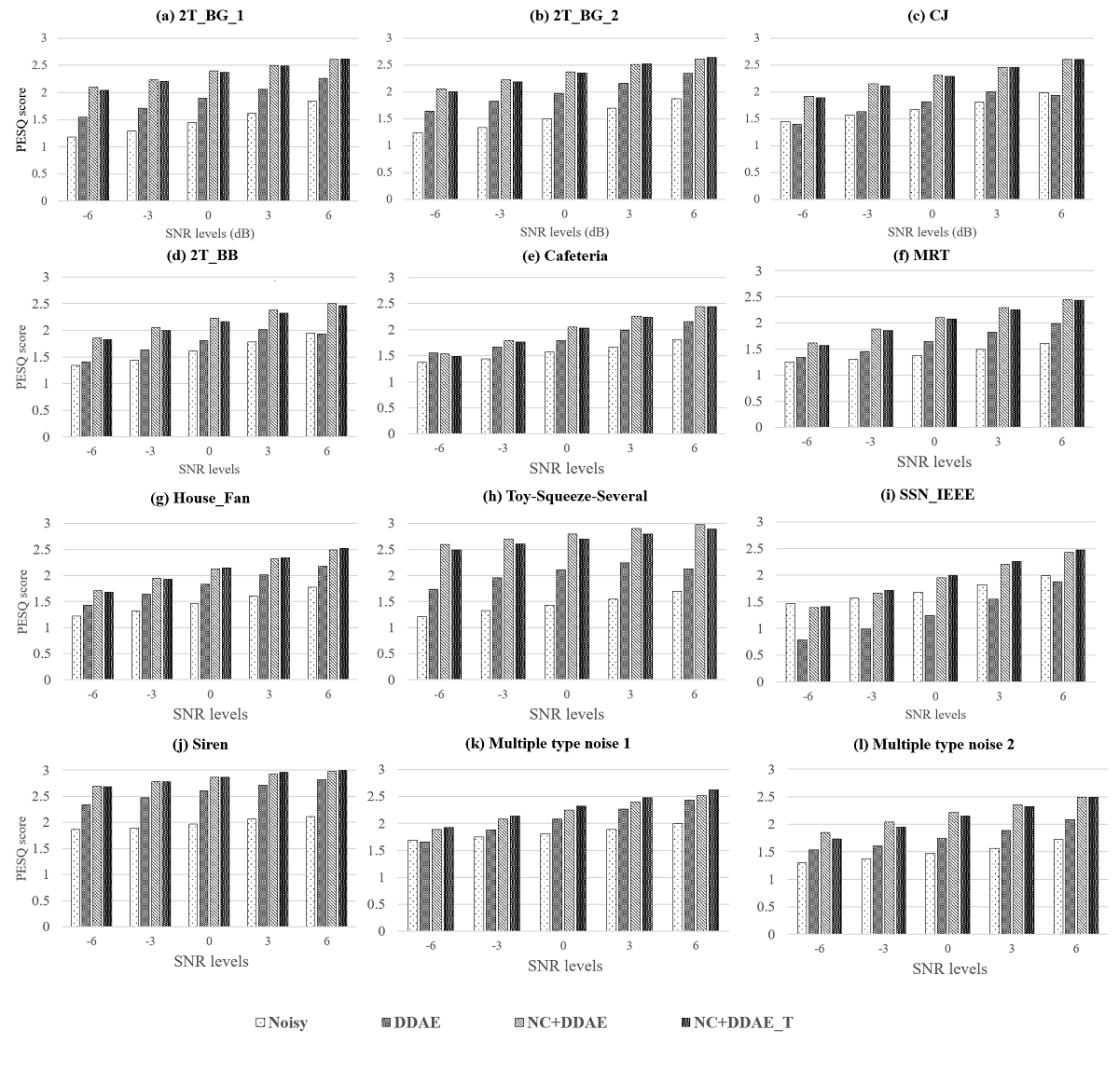
Mean perceptual evaluation of speech quality (PESQ) scores of the 4 noise reduction approaches. 2T_BB: 2 Talker; 2T_BG_1: 2 talker_unseen1; 2T_BG_2: 2 talker_unseen2; CJ: Construction Jackhammer; dB: decibel; DDAE: deep denoising autoencoder; NC: noise classifier; NC+DDAE_T: noise classifier + deep denoising autoencoder with knowledge transfer; MRT: Mass Rapid Transit; PESQ: perceptual evaluation of speech quality; SNR: signal-to-noise ratio; SSN_IEEE: speech shape noise from the Institute of Electrical and Electronics Engineers.

**Figure 5:**
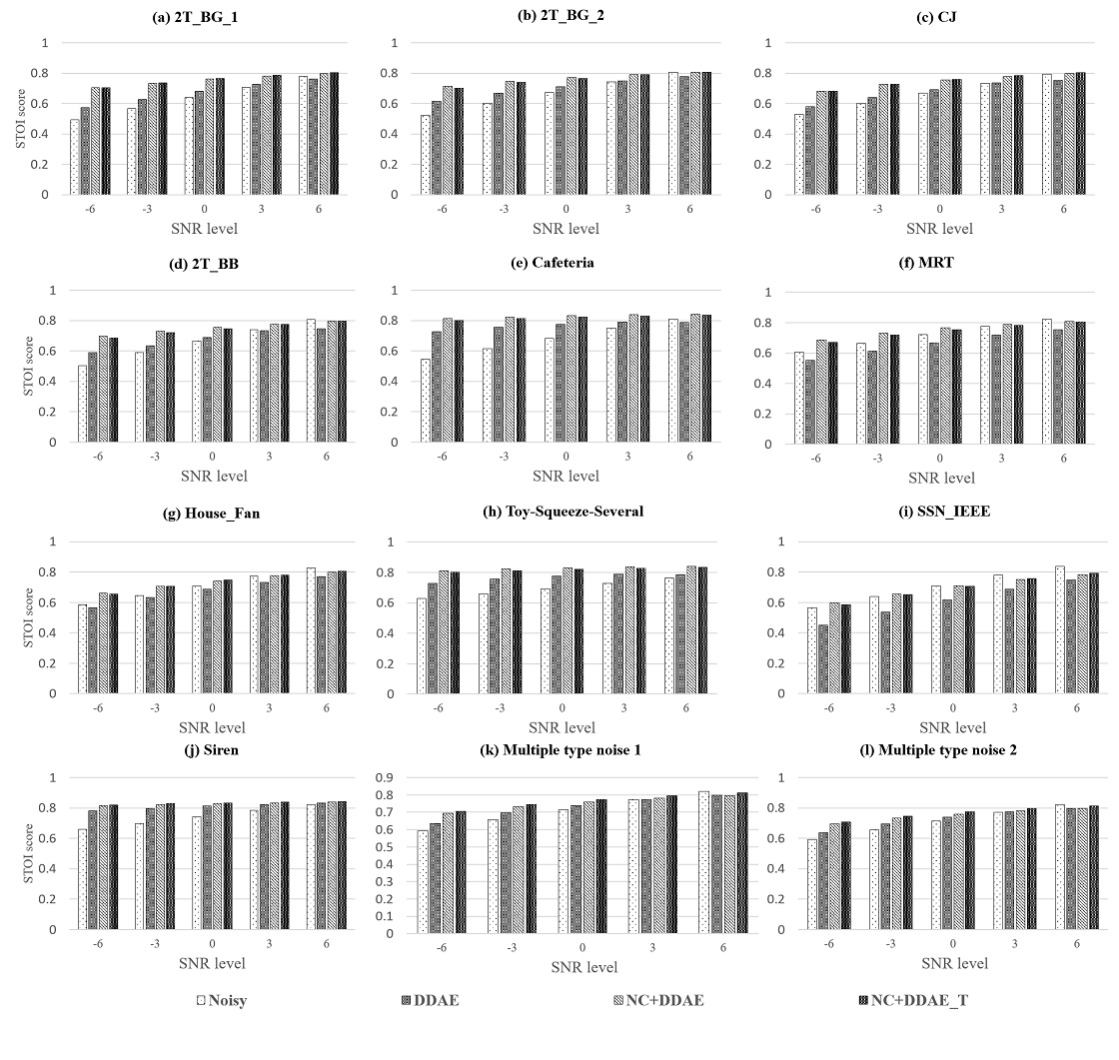
Mean short-time objective intelligibility (STOI) scores of the different noise reduction approaches. 2T_BB: 2 Talker; 2T_BG_1: 2 talker_unseen1; 2T_BG_2: 2 talker_unseen2; CJ: Construction Jackhammer; DDAE: deep denoising autoencoder; NC: noise classifier; NC+DDAE_T: noise classifier + deep denoising autoencoder with knowledge transfer; MRT: Mass Rapid Transit; SNR: signal-to-noise ratio; SSN_IEEE: speech shape noise from the Institute of Electrical and Electronics Engineers; STOI: short-time objective intelligibility.

We compared the newly proposed NC+DDAE_T with the previously established NR systems, DDAE and NC+DDAE. The PESQ and STOI scores of these tests are shown in Figures 4 and 5, respectively. As demonstrated in Figure 4, the PESQ scores of the proposed NC+DDAE_T are generally similar to those of the NC+DDAE. The details regarding the average scores of each approach (ie, noisy, DDAE, NC+DDAE, and NC+DDAE_T) for the 12 background noises at 6 different SNR levels can be found in Table A1 of Multimedia Appendix 4. In the STOI scores, the NC+DDAE_T model also achieved the same level as did the NC+DDAE (Figure 5). The detailed STOI scores are listed in Table A2 of Multimedia Appendix 4. These objective evaluation results proved that the NC+DDAE_T could provide almost the same speech intelligibility performance as the NC+DDAE.

### Recognition in Listening Tests

**Figure 6:**
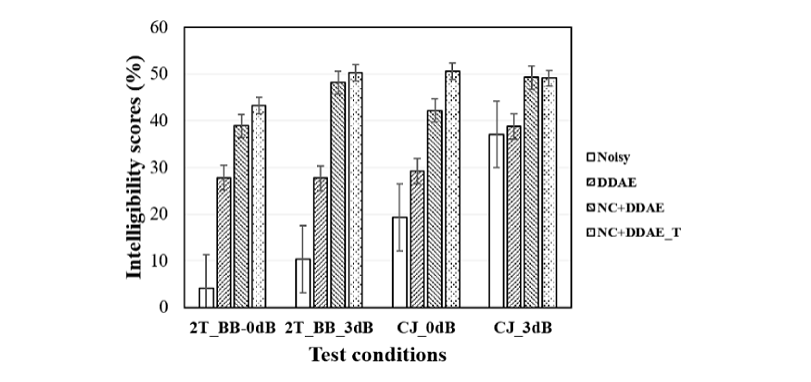
Mean intelligibility scores of 10 participants with cochlear implants in 4 types of simulated test conditions. 2T_BB: 2 Talker; CJ: Construction Jackhammer; dB: decibel; DDAE: deep denoising autoencoder; NC: noise classifier; NC+DDAE_T: noise classifier + deep denoising autoencoder with knowledge transfer.

Figure 6 shows the average WCR scores of 10 individuals with CI in the 2 Talker and CJ noise conditions each at 0- and 3-dB SNR levels. The detailed results are as follows: The respective average WCR scores and standard error of the mean (SEM) for noisy, DDAE, NC+DDAE, and NC+DDAE_T with 2 Talker background noise were 4.1 (SEM 1.87), 27.8 (SEM 5.42), 38.9 (SEM 8.83), and 43.2 (SEM 9.33) at the 0-dB SNR level; and 10.3 (SEM 3.84), 27.7 (SEM 5.24), 48.2 (SEM 9.69), and 50.3 (SEM 8.98) at the 3-dB SNR level. In the CJ background noise, the respective average scores and SEMs were 19.3 (SEM 5.76), 27.7 (SEM 5.24), 42.2 (SEM 9.64), and 50.6 (SEM 10.0) at the 0-dB SNR level; and 37.1 (SEM 9.84), 38.8 (SEM 8.41), 49.3 (SEM9.31), and 50.9 (SEM 10.13) at the 3-dB SNR level. These results demonstrated that the NC+DDAE_T provided better speech intelligibility scores than did noisy speech. Moreover, the newly developed NC+DDAE_T model achieved slightly higher intelligibility performances than did the NC+DDAE approach under most test conditions. The 1-way analysis of variance (ANOVA) [44] with least significant difference post hoc comparison [45] was used to analyze the results of the 4 NR systems (noisy, DDAE, NC+DDAE, and NC+DDAE_T) in the 4 test conditions. The 1-way ANOVA result confirmed that the WCR scores differed significantly among the 4 systems (*F*=13.256; *P*<.001). The least significant difference post hoc comparisons (Table 1) further revealed that the noisy condition was significantly different from the other 3 systems (DDAE: *P*=.16; NC+DDAE: *P*<.001; NC+DDAE_T: *P*<.001). Meanwhile, the differences between the NC+DDAE and NC+DDAE_T models were not significant (*P*=.50).

**Table 1:** The mean difference, standard error, and significance of the listening test in each noise reduction system.

Method (I) by test (J)			Mean difference (I–J) (standard error)		*P* value^a^
**Noisy (I)**					
	DDAE^b^ (J)	–13.18 (5.428)		*.016* ^c^	
	NC^d^+DDAE (J)	–26.95 (5.428)		*<* *.001*	
	NC+DDAE_T^e^ (J)	–30.60 (5.428)		*<* *.001*	
**DDAE (I)**					
	Noisy (J)	13.18 (5.428)		*.* *02*	
	NC+DDAE (J)	–13.78 (5.428)		*.01*	
	NC+DDAE_T (J)	–17.43 (5.428)		*.002*	
**NC+DDAE (I)**					
	Noisy (J)	26.95 (5.428)		*<* *.001*	
	DDAE (J)	13.78 (5.428)		*.01*	
	NC+DDAE_T (J)	–3.65 (5.428)		.50	
**NC+DDAE_T (I)**					
	Noisy (J)	30.60 (5.428)		*<* *.001*	
	DDAE (J)	17.43 (5.428)		*.* *002*	
	NC+DDAE (J)	3.65 (5.428)		.50	

^a^*P* values are significant at α = .05. Least significant difference was selected to conduct post hoc testing.

^b^DDAE: deep denoising autoencoder.

^c^Values in italics represent significant values.

^d^NC: noise classifier.

^e^NC+DDAE_T: noise classifier + deep denoising autoencoder with knowledge transfer.

### Comparison of the Numbers of Parameters

The original structure of the NC+DDAE system used 12 ND+DDAEs and 1 NI+DDAE for the NR. In this study, the newly developed NC+DDAE_T system only needed 1 NI+DDAE and 12 different layer parameters to achieve the same performance as the previous NC+DDAE system. We further compared the numbers of parameters between the NC+DDAE and NC+DDAE_T approaches. The NC+DDAE_T approach required only 0.1 million parameters while the previous NC+DDAE system needed 4.4 million parameters. The number of parameters was thus reduced by 76.5% compared to the previous approach.

## Discussion

### Layers for Substitution

This study proposed a new NC+DDAE_T NR model that helps CI users to improve speech intelligibility in noisy listening conditions. Knowledge transfer technology was used to reduce the parameter requirements in comparison to the previous NC+DDAE approach. The experimental results of the objective evaluation and the subjective listening tests demonstrated that the NC+DDAE_T achieved performances comparable to those of the NC+DDAE approach, while the number of parameters used by the NC+DDAE_T was reduced by 76.5% compared to the NC+DDAE. Therefore, knowledge transfer technology could be a useful approach to further improve the benefits of NC+DDAE in reducing the cost of implementation in the future.

**Figure 7:**
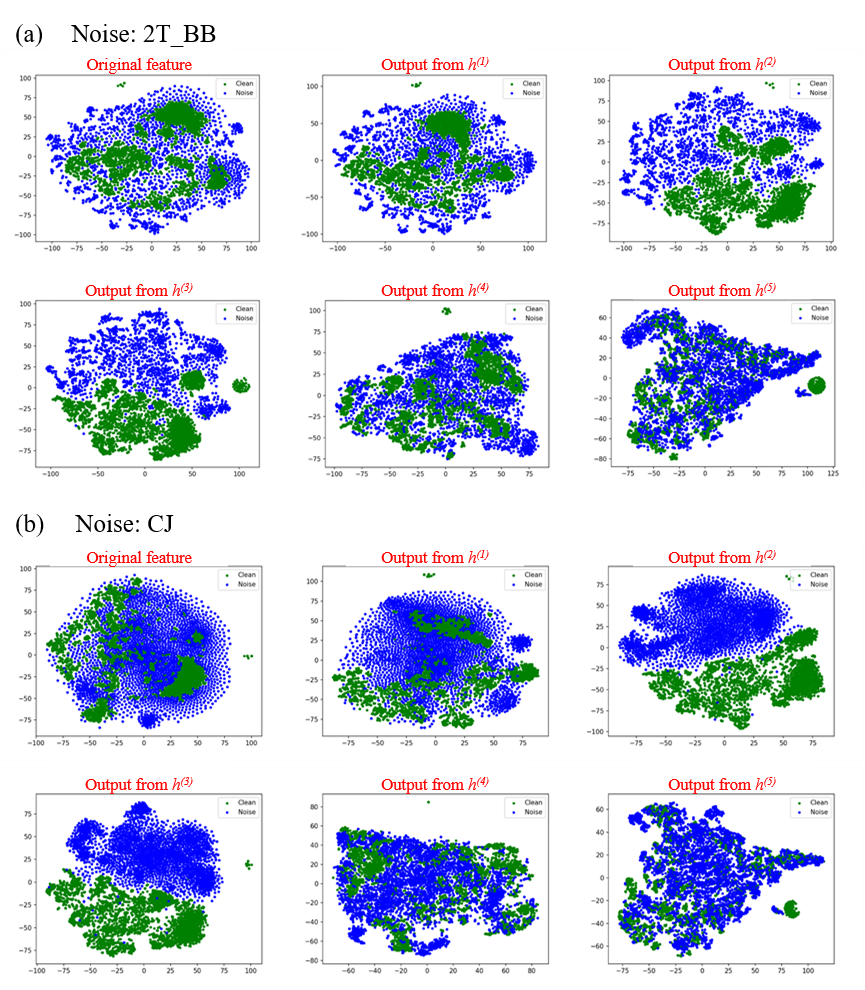
*t*-distributed stochastic neighbor embedding (*t*-SNE) feature analysis of each layer in the noise-independent deep denoising autoencoder (NI-DDAE) model with noisy and clean speech data. The green dots represent the output features of clean speech and the blue dots indicate features of noisy speech. 2T_BB: 2 Talker; CJ: Construction Jackhammer.

The architecture of the NC+DDAE_T, (ie, which layer is substituted) is the basis for achieving higher performance with this novel system compared to the NC-DDAE. According to the objective evaluation by PESQ and STOI scores (Multimedia Appendix 1), the substitution of the middle layer can achieve better performances. To further analyze why the middle layer was so important, *t*-distributed stochastic neighbor embedding (*t*-SNE) [46] was used to visualize the features that output by each layer. The acoustic features of noisy and clean speech (ie, LPS) were the inputs for the trained NI-DDAE NR model. The output features of each NI-DDAE layer were analyzed using *t*-SNE, which can project the distribution of each layer onto a 2D plane. Figure 7 shows the results of this feature visualization. Green dots represent the output features of clean speech, whereas blue dots indicate features of noisy speech. The less overlap is apparent between the green and blue areas, the better the layer can separate the features. These results indicate that clean and noisy data were primarily separated in the output from h^(2)^ and h^(3)^, implying that the front layers help to distinguish noisy speech from clean features and thus could be the most important layers. This interpretation is also consistent with the objective evaluation results in Multimedia Appendix 1.

To explain the phenomenon illustrated in Figure 7, we suggest that the NC+DDAE_T model may work similarly to the human brain. The first layers of the model may try to separate the noise from the speech features. Therefore, these features would diverge completely in the middle layers of this NR model. The model would then try to reconstruct the enhanced speech and lower the volume of the noise in the final layers of the model; hence, the features would converge again in the *t*-SNE analysis. Based on these hypotheses, the second layer may be the key to feature separation because the features are well separated after the second layer. Therefore, to adapt the NR model to a specific type of noise, substituting the second layer would be the best choice, which corresponds to the results of the objective evaluation. The other parts of the NC+DDAE_T model may work as preprocessing and vocoder units. These parts are common units of all NR models; thus, different ND-DDAEs can share the same weight and bias values. Therefore, the concept of knowledge transfer can be used in this part to decrease the size of each model.

### Future Perspectives

**Figure 8:**
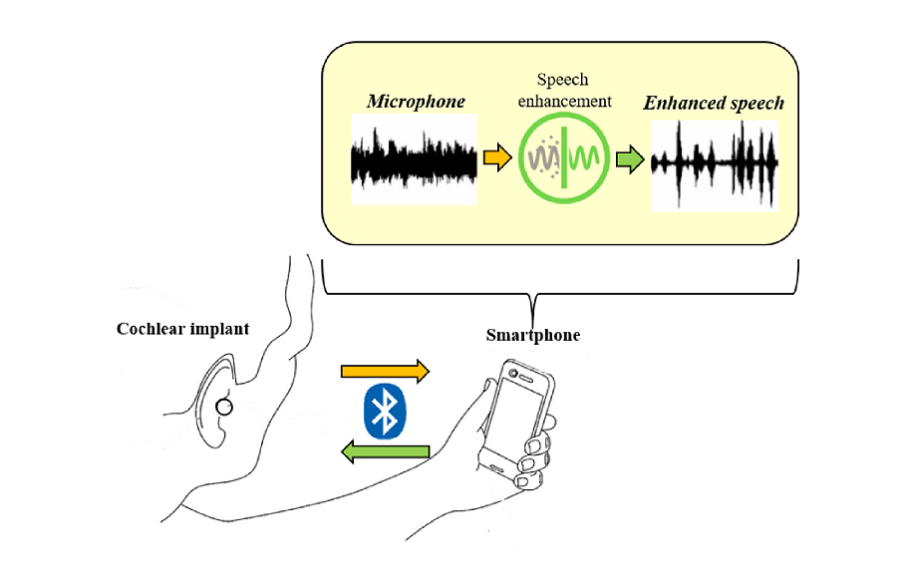
Schematic of the noise classifier deep denoising autoencoder with knowledge transfer (NC+DDAE_T) implementation.

Based on previous and current results of objective evaluation and listening tests, we can conclude that the proposed NC+DDAE_T performs comparably to the NC+DDAE. In addition, the NC+DDAE_T needs only a quarter of the number of parameters compared to the 12 ND-DDAE models. These characteristics suggest a great potential for future implementation of the NC+DDAE_T model. With the decreased number of parameters, an implemented device would require less memory. To prove this concept, we have implemented the NC+DDAE_T architecture in an app on an iPhone XR mobile phone (Apple Inc) as shown in Figure 8. The processing time could satisfy the maximum group delay requirement of assistive listening devices. With this advantage of edge computing, the proposed NC+DDAE_T may become a new kind of hearing assistive technology in the near future.

### Limitations

The proposed NC+DDAE_T is an adaptable NR system, which means that the system benefits may be affected by the training data (eg, background noise types, speakers). Therefore, if the proposed system faces noisy conditions that are very different from the training data (ie, mismatch conditions), the proposed system would require major improvements, and new recordings of noise data may be needed. Overcoming this issue requires future study. Additionally, although the proposed system was implemented in an app, the full implementation of the proposed system in the hardware of currently used CI devices is still a way off. However, as studies increasingly focus on the acceleration of DL-based models in microprocessors [47,48], there is a greater chance that DL technologies may be implemented into CI devices in the near future.

### Conclusions

This study proposed a novel NC+DDAE_T system for NR in CI devices. The knowledge transfer approach was used to lower the number of parameters of the DDAE model. The experimental results of the objective evaluations, along with the listening tests, showed that the proposed NC+DDAE_T model provided comparable performance to the previously established NC+DDAE NR model. These results suggest that the proposed NC+DDAE_T model may be a new NR system that can enable CI users to hear well in noisy conditions.

## Appendix

Multimedia Appendix 1 Results Following Replacement of Each Layer of weight and bias of the Deep Denoising Autoencoder Model.

Multimedia Appendix 2 Confusion matrix of the 12 noise classifications.

Multimedia Appendix 3 Individual biographical data of the attended cochlear implant subjects.

Multimedia Appendix 4 Perceptual evaluation of speech quality and short-time objective intelligibility scores of different noise reduction systems.

## Abbreviations

ANOVA: analysis of variance
CI: cochlear implant
CJ: Construction Jackhammer
DDAE: deep denoising autoencoder
DL: deep learning
DNN: deep neural network
LPS: log power spectra
MRT: Mass Rapid Transit
NC: noise classifier
NC+DDAE_T: noise classifier + deep denoising autoencoder with knowledge transfer
ND: noise-dependent
NI: noise-independent
NR: noise reduction
PESQ: perceptual evaluation of speech quality
SEM: standard error of the mean
SNR: signal-to-noise ratio
SSN_IEEE: speech shape noise from the Institute of Electrical and Electronics Engineers
STOI: short-time objective intelligibility
TMHINT: Taiwan Mandarin version of the hearing in noise test
*t*-SNE: *t*-distributed stochastic neighbor embedding
WCR: word correct rate
