# Supplementary material for: Improved Environment-Aware–Based Noise Reduction System for Cochlear Implant Users Based on a Knowledge Transfer Approach: Development and Usability Study
Source: J Med Internet Res. 2021 Oct 28;23(10):e25460. doi: 10.2196/25460 (PMC8587190; doi:10.2196/25460)
Supplement: Multimedia Appendix 1 [file jmir_v23i10e25460_app1.docx]

## Appendix 1. Pilot Study: Results Following Replacement of Each Layer of weight and bias of the Deep Denoising Autoencoder Model

This section shows the benefits of substituting each layer of weight and bias in the noise classifier deep denoising autoencoder with knowledge transfer (NC+DDAE_T)*.* In Fig. A1, the $T\left( 1 \right)$ denotes weight and bias of left part of *first hidden* layer ($h^{(1)}$); and the $T\left( 2 \right)$ to $T\left( 6 \right)$ were the weight and bias of right part of $h^{(1)}$ to $h^{(5)}$, respectively. The PESQ and STOI scores were used to evaluate the performances of the models that substituting each layer of weight and bias ($T\left( 1 \right) \sim T(6)$). Tables A3 and A4 show the detailed values of Figures A2 and A3, respectively.

**Figure A1.** Schematic of the weight and bias $T\left( 1 \right)$ to$T(6)$ in the noise-independent deep denoising autoencoder (NI-DDAE).

**Figure A2.** Perceptual evaluation of speech quality (PESQ) scores when substituting individual layer of weight and bias of the noise classifier deep denoising autoencoder with knowledge transfer (NC+DDAE_T).

**Figure A3.** STOI scores when substituting individual layer of weight and bias of the noise classifier deep denoising autoencoder with knowledge transfer (NC+DDAE_T).

| **Table A3.** Perceptual evaluation of speech quality (PESQ) scores of each substituted model. | | | | | | |
| --- | --- | --- | --- | --- | --- | --- |
| Noise type | Substituting layer | SNR levels | | | | |
|  |  | −6 dB | −3 dB | 0 dB | 3 dB | 6 dB |
| 2T_BG_1 | NC+DDAE_T(1) | 1.96081 | 2.12777 | 2.2974 | 2.41522 | 2.55363 |
|  | NC+DDAE_T(2) | **2.06295** | **2.23355** | **2.39853** | **2.50806** | **2.63125** |
|  | NC+DDAE_T(3) | 2.04021 | 2.20953 | 2.37429 | 2.49191 | 2.61650 |
|  | NC+DDAE_T(4) | 1.97537 | 2.13935 | 2.30740 | 2.42768 | 2.56115 |
|  | NC+DDAE_T(5) | 1.79924 | 1.97772 | 2.16276 | 2.30635 | 2.42972 |
|  | NC+DDAE_T(6) | 1.93274 | 2.17394 | 2.34016 | 2.49265 | 2.63841 |
| 2T_BG_2 | NC+DDAE_T(1) | 1.94021 | 2.1249 | 2.28607 | 2.43343 | 2.57048 |
|  | NC+DDAE_T(2) | **2.03331** | **2.20868** | **2.37624** | 2.51441 | 2.63960 |
|  | NC+DDAE_T(3) | 2.00445 | 2.18846 | 2.35237 | **2.51763** | **2.64091** |
|  | NC+DDAE_T(4) | 1.93750 | 2.10750 | 2.28263 | 2.44002 | 2.56376 |
|  | NC+DDAE_T(5) | 1.74176 | 1.94938 | 2.12818 | 2.29230 | 2.42258 |
|  | NC+DDAE_T(6) | 1.55859 | 1.67085 | 1.82063 | 1.99385 | 2.13938 |
| CJ | NC+DDAE_T(1) | 1.80864 | 2.04342 | 2.21369 | 2.38749 | 2.54246 |
|  | NC+DDAE_T(2) | **1.90264** | **2.12485** | **2.29125** | **2.46103** | 2.60342 |
|  | NC+DDAE_T(3) | 1.88731 | 2.11390 | 2.29116 | 2.45419 | **2.60821** |
|  | NC+DDAE_T(4) | 1.79439 | 2.03445 | 2.21656 | 2.38385 | 2.54399 |
|  | NC+DDAE_T(5) | 1.66092 | 1.90876 | 2.09506 | 2.27938 | 2.45005 |
|  | NC+DDAE_T(6) | 1.91722 | 2.15437 | 2.30723 | 2.45712 | 2.60721 |
| 2T_BB | NC+DDAE_T(1) | 1.70995 | 1.87422 | 2.05351 | 2.21895 | 2.36238 |
|  | NC+DDAE_T(2) | 1.82594 | **1.99469** | 2.15763 | **2.32740** | **2.46192** |
|  | NC+DDAE_T(3) | **1.82756** | 1.99451 | **2.16505** | 2.32402 | 2.4618 |
|  | NC+DDAE_T(4) | 1.71462 | 1.89772 | 2.07724 | 2.23529 | 2.37760 |
|  | NC+DDAE_T(5) | 1.54963 | 1.71589 | 1.87803 | 2.03839 | 2.1865 |
|  | NC+DDAE_T(6) | 1.29673 | 1.42591 | 1.57894 | 1.71649 | 1.84666 |
| MRT | NC+DDAE_T(1) | 1.54227 | 1.80046 | 2.03506 | 2.22630 | 2.40993 |
|  | NC+DDAE_T(2) | **1.61997** | **1.86942** | **2.09901** | **2.28497** | **2.45804** |
|  | NC+DDAE_T(3) | 1.56823 | 1.85193 | 2.07191 | 2.25727 | 2.43719 |
|  | NC+DDAE_T(4) | 1.49332 | 1.76397 | 2.00545 | 2.20823 | 2.38220 |
|  | NC+DDAE_T(5) | 1.37402 | 1.65124 | 1.91867 | 2.13116 | 2.31424 |
|  | NC+DDAE_T(6) | 1.16129 | 1.37137 | 1.57937 | 1.77110 | 1.93143 |
| Cafeteria | NC+DDAE_T(1) | 1.45739 | 1.7179 | 1.98503 | 2.20772 | 2.40352 |
|  | NC+DDAE_T(2) | **1.53510** | **1.8075** | **2.06521** | **2.26496** | **2.45863** |
|  | NC+DDAE_T(3) | 1.48630 | 1.75854 | 2.03395 | 2.24431 | 2.44498 |
|  | NC+DDAE_T(4) | 1.42001 | 1.70304 | 1.97639 | 2.19580 | 2.40190 |
|  | NC+DDAE_T(5) | 1.31199 | 1.59723 | 1.88343 | 2.12892 | 2.34554 |
|  | NC+DDAE_T(6) | 1.20912 | 1.42815 | 1.62715 | 1.79837 | 1.96229 |
| House-Fan | NC+DDAE_T(1) | 1.62504 | 1.87250 | 2.07446 | 2.26981 | 2.46264 |
|  | NC+DDAE_T(2) | **1.71598** | **1.95420** | **2.16539** | **2.36959** | **2.53613** |
|  | NC+DDAE_T(3) | 1.67674 | 1.92679 | 2.14520 | 2.34080 | 2.52155 |
|  | NC+DDAE_T(4) | 1.58794 | 1.85767 | 2.07688 | 2.28907 | 2.47734 |
|  | NC+DDAE_T(5) | 1.46655 | 1.72827 | 1.95836 | 2.17629 | 2.37604 |
|  | NC+DDAE_T(6) | 1.36838 | 1.59451 | 1.77707 | 1.91703 | 2.07200 |
| Toy | NC+DDAE_T(1) | 2.46919 | 2.57650 | 2.66797 | 2.75674 | 2.84778 |
|  | NC+DDAE_T(2) | **2.53045** | **2.63325** | **2.73150** | **2.81913** | **2.90875** |
|  | NC+DDAE_T(3) | 2.49402 | 2.60439 | 2.70511 | 2.79698 | 2.89269 |
|  | NC+DDAE_T(4) | 2.42620 | 2.53137 | 2.63925 | 2.73421 | 2.83392 |
|  | NC+DDAE_T(5) | 2.30647 | 2.41829 | 2.52597 | 2.63572 | 2.72851 |
|  | NC+DDAE_T(6) | 1.99509 | 2.22907 | 2.38619 | 2.52587 | 2.66883 |
| SSN_IEEE | NC+DDAE_T(1) | 1.43666 | 1.73015 | 1.96022 | 2.20047 | 2.41986 |
|  | NC+DDAE_T(2) | **1.49787** | **1.79144** | **2.05087** | **2.27440** | **2.49345** |
|  | NC+DDAE_T(3) | 1.41179 | 1.71474 | 1.99672 | 2.25580 | 2.47390 |
|  | NC+DDAE_T(4) | 1.30935 | 1.61682 | 1.90993 | 2.17904 | 2.41078 |
|  | NC+DDAE_T(5) | 1.26360 | 1.53485 | 1.79625 | 2.04979 | 2.28747 |
|  | NC+DDAE_T(6) | 1.33090 | 1.58351 | 1.79335 | 1.97611 | 2.11241 |
| Siren | NC+DDAE_T(1) | 2.67518 | 2.76140 | 2.87149 | 2.94567 | 3.01902 |
|  | NC+DDAE_T(2) | **2.72682** | **2.81373** | **2.91680** | **2.98902** | **3.06643** |
|  | NC+DDAE_T(3) | 2.68787 | 2.78010 | 2.86935 | 2.96171 | 3.03783 |
|  | NC+DDAE_T(4) | 2.62854 | 2.72368 | 2.83340 | 2.92014 | 3.00287 |
|  | NC+DDAE_T(5) | 2.53445 | 2.63225 | 2.73806 | 2.83466 | 2.90159 |
|  | NC+DDAE_T(6) | 2.40694 | 2.50855 | 2.61885 | 2.68901 | 2.76176 |
| Multiple type noise 1 | NC+DDAE_T(1) | 1.89009 | 2.10816 | 2.29363 | 2.45553 | 2.60098 |
|  | NC+DDAE_T(2) | **1.94692** | **2.15561** | **2.33727** | **2.49012** | **2.63599** |
|  | NC+DDAE_T(3) | 1.92933 | 2.13866 | 2.32350 | 2.48244 | 2.6223 |
|  | NC+DDAE_T(4) | 1.87569 | 2.08186 | 2.26833 | 2.44952 | 2.59654 |
|  | NC+DDAE_T(5) | 1.75652 | 1.99312 | 2.19368 | 2.37403 | 2.52728 |
|  | NC+DDAE_T(6) | 1.65612 | 1.88000 | 2.05000 | 2.21330 | 2.33448 |
| Multiple type noise 2 | NC+DDAE_T(1) | 1.66592 | 1.88061 | 2.09468 | 2.28003 | 2.44075 |
|  | NC+DDAE_T(2) | **1.78033** | **1.97980** | **2.17557** | **2.35129** | **2.50920** |
|  | NC+DDAE_T(3) | 1.73647 | 1.95311 | 2.15381 | 2.32559 | 2.49223 |
|  | NC+DDAE_T(4) | 1.59146 | 1.82964 | 2.04756 | 2.23259 | 2.41594 |
|  | NC+DDAE_T(5) | 1.43411 | 1.68129 | 1.93022 | 2.12402 | 2.30538 |
|  | NC+DDAE_T(6) | 1.14745 | 1.40801 | 1.61980 | 1.78921 | 1.94304 |

| **Table A4.** Short-time objective intelligibility (STOI) scores of each substituted model. | | | | | | |
| --- | --- | --- | --- | --- | --- | --- |
| Noise type | Substituting layer | SNR levels | | | | |
|  |  | −6 dB | −3 dB | 0 dB | 3 dB | 6 dB |
| 2T_BG_1 | NC+DDAE_T(1) | 0.68722 | 0.72363 | 0.75138 | 0.77479 | 0.79374 |
|  | NC+DDAE_T(2) | **0.70463** | **0.73833** | **0.76554** | 0.78704 | 0.80334 |
|  | NC+DDAE_T(3) | 0.70317 | 0.73766 | 0.76525 | **0.78708** | **0.80343** |
|  | NC+DDAE_T(4) | 0.68308 | 0.71905 | 0.75040 | 0.77376 | 0.79189 |
|  | NC+DDAE_T(5) | 0.6212 | 0.66427 | 0.69805 | 0.72937 | 0.75197 |
|  | NC+DDAE_T(6) | 0.67858 | 0.72329 | 0.75447 | 0.77947 | 0.79677 |
| 2T_BG_2 | NC+DDAE_T(1) | 0.68847 | 0.72676 | 0.75543 | 0.77890 | 0.79656 |
|  | NC+DDAE_T(2) | **0.70501** | **0.74029** | **0.76747** | 0.78865 | 0.80500 |
|  | NC+DDAE_T(3) | 0.70212 | 0.73931 | 0.76617 | **0.78940** | **0.80667** |
|  | NC+DDAE_T(4) | 0.68113 | 0.72045 | 0.74896 | 0.77415 | 0.79361 |
|  | NC+DDAE_T(5) | 0.62098 | 0.66877 | 0.70248 | 0.73285 | 0.76755 |
|  | NC+DDAE_T(6) | 0.55808 | 0.61703 | 0.66581 | 0.71084 | 0.74762 |
| CJ | NC+DDAE_T(1) | 0.66352 | 0.71048 | 0.74458 | 0.77088 | 0.79120 |
|  | NC+DDAE_T(2) | **0.68405** | **0.72716** | 0.75754 | 0.78367 | 0.80169 |
|  | NC+DDAE_T(3) | 0.68169 | 0.72632 | **0.75809** | **0.78434** | **0.80394** |
|  | NC+DDAE_T(4) | 0.65716 | 0.70572 | 0.74243 | 0.77062 | 0.79286 |
|  | NC+DDAE_T(5) | 0.60918 | 0.66218 | 0.70309 | 0.73443 | 0.76349 |
|  | NC+DDAE_T(6) | 0.68171 | 0.72536 | 0.75494 | 0.77922 | 0.79585 |

| 2T_BB | NC+DDAE_T(1) | 0.66285 | 0.70151 | 0.73073 | 0.76107 | 0.78337 |
| --- | --- | --- | --- | --- | --- | --- |
|  | NC+DDAE_T(2) | 0.68258 | 0.71832 | 0.74582 | **0.77275** | 0.79246 |
|  | NC+DDAE_T(3) | **0.68397** | **0.72053** | **0.74728** | 0.77458 | **0.79637** |
|  | NC+DDAE_T(4) | 0.65240 | 0.69433 | 0.72406 | 0.75393 | 0.78011 |
|  | NC+DDAE_T(5) | 0.59422 | 0.63475 | 0.67019 | 0.70006 | 0.73023 |
|  | NC+DDAE_T(6) | 0.52301 | 0.56700 | 0.59912 | 0.62643 | 0.64764 |
| MRT | NC+DDAE_T(1) | 0.66346 | 0.70971 | 0.74856 | 0.77655 | 0.79787 |
|  | NC+DDAE_T(2) | **0.67663** | **0.72144** | **0.75626** | **0.78269** | **0.80306** |
|  | NC+DDAE_T(3) | 0.67017 | 0.71860 | 0.75492 | 0.78151 | 0.80298 |
|  | NC+DDAE_T(4) | 0.63930 | 0.69388 | 0.73765 | 0.76936 | 0.79162 |
|  | NC+DDAE_T(5) | 0.59785 | 0.66052 | 0.70898 | 0.74855 | 0.77397 |
|  | NC+DDAE_T(6) | 0.54970 | 0.60034 | 0.63673 | 0.66460 | 0.68569 |
| Cafeteria | NC+DDAE_T(1) | 0.79851 | 0.80896 | 0.81820 | 0.82625 | 0.83253 |
|  | NC+DDAE_T(2) | **0.80557** | **0.81527** | **0.82411** | **0.83122** | **0.83670** |
|  | NC+DDAE_T(3) | 0.80080 | 0.81241 | 0.82168 | 0.82870 | 0.83504 |
|  | NC+DDAE_T(4) | 0.78627 | 0.80002 | 0.81082 | 0.81955 | 0.82671 |
|  | NC+DDAE_T(5) | 0.74860 | 0.76869 | 0.78335 | 0.79729 | 0.80707 |
|  | NC+DDAE_T(6) | 0.70500 | 0.74346 | 0.76836 | 0.78818 | 0.80217 |
| House-Fan | NC+DDAE_T(1) | 0.64552 | 0.69580 | 0.73782 | 0.76964 | 0.79394 |
|  | NC+DDAE_T(2) | **0.66416** | **0.71152** | 0.74640 | **0.78066** | 0.80122 |
|  | NC+DDAE_T(3) | 0.65702 | 0.70742 | **0.74788** | 0.78003 | **0.80444** |
|  | NC+DDAE_T(4) | 0.62423 | 0.67883 | 0.72648 | 0.76488 | 0.79284 |
|  | NC+DDAE_T(5) | 0.58754 | 0.64185 | 0.69394 | 0.73840 | 0.77252 |
|  | NC+DDAE_T(6) | 0.55051 | 0.60093 | 0.64017 | 0.66679 | 0.68988 |

| Toy | NC+DDAE_T(1) | 0.79851 | 0.80896 | 0.81820 | 0.82625 | 0.83253 |
| --- | --- | --- | --- | --- | --- | --- |
|  | NC+DDAE_T(2) | **0.80557** | **0.81527** | **0.82411** | **0.83122** | 0.83670 |
|  | NC+DDAE_T(3) | 0.80080 | 0.81241 | 0.82168 | 0.82870 | **0.83504** |
|  | NC+DDAE_T(4) | 0.78627 | 0.80002 | 0.81082 | 0.81955 | 0.82671 |
|  | NC+DDAE_T(5) | 0.74860 | 0.76869 | 0.78335 | 0.79729 | 0.80707 |
|  | NC+DDAE_T(6) | 0.70500 | 0.74346 | 0.76836 | 0.78818 | 0.80217 |
| SSN_IEEE | NC+DDAE_T(1) | 0.56979 | 0.63979 | 0.69397 | 0.74538 | 0.77937 |
|  | NC+DDAE_T(2) | **0.59082** | **0.65562** | **0.70939** | 0.75510 | 0.78817 |
|  | NC+DDAE_T(3) | 0.58422 | 0.65132 | 0.70818 | **0.75948** | **0.79360** |
|  | NC+DDAE_T(4) | 0.56072 | 0.63299 | 0.69245 | 0.74672 | 0.78389 |
|  | NC+DDAE_T(5) | 0.51928 | 0.59373 | 0.65563 | 0.71037 | 0.75311 |
|  | NC+DDAE_T(6) | 0.51156 | 0.57368 | 0.62471 | 0.66897 | 0.70214 |
| Siren | NC+DDAE_T(1) | 0.81777 | 0.82623 | 0.83287 | 0.83817 | 0.84163 |
|  | NC+DDAE_T(2) | **0.82180** | **0.82852** | **0.83537** | 0.83931 | 0.84301 |
|  | NC+DDAE_T(3) | 0.81880 | 0.82728 | 0.83356 | **0.83940** | **0.84302** |
|  | NC+DDAE_T(4) | 0.80892 | 0.81960 | 0.82742 | 0.83316 | 0.83868 |
|  | NC+DDAE_T(5) | 0.78767 | 0.80054 | 0.81268 | 0.81962 | 0.82612 |
|  | NC+DDAE_T(6) | 0.77863 | 0.78886 | 0.79705 | 0.80310 | 0.80835 |
| Multiple type noise 1 | NC+DDAE_T(1) | 0.69424 | 0.73560 | 0.76533 | 0.78999 | 0.80493 |
|  | NC+DDAE_T(2) | **0.70797** | 0.74554 | 0.77280 | 0.79504 | 0.80999 |
|  | NC+DDAE_T(3) | 0.70613 | **0.74561** | **0.77331** | **0.79639** | **0.81233** |
|  | NC+DDAE_T(4) | 0.68455 | 0.72947 | 0.76125 | 0.78806 | 0.80648 |
|  | NC+DDAE_T(5) | 0.63997 | 0.69589 | 0.73659 | 0.76865 | 0.7893 |
|  | NC+DDAE_T(6) | 0.60535 | 0.65507 | 0.68592 | 0.70869 | 0.72546 |

| Multiple type 2 noise 2 | NC+DDAE_T(1) | 0.68284 | 0.72285 | 0.75522 | 0.78102 | 0.79908 |
| --- | --- | --- | --- | --- | --- | --- |
|  | NC+DDAE_T(2) | **0.70191** | **0.73589** | **0.76452** | **0.78819** | 0.80534 |
|  | NC+DDAE_T(3) | 0.69558 | 0.73256 | 0.76349 | 0.78654 | **0.80563** |
|  | NC+DDAE_T(4) | 0.64574 | 0.69287 | 0.73423 | 0.76768 | 0.79288 |
|  | NC+DDAE_T(5) | 0.59422 | 0.64865 | 0.69972 | 0.73673 | 0.76753 |
|  | NC+DDAE_T(6) | 0.55776 | 0.61189 | 0.65240 | 0.68339 | 0.70613 |
