# Supplementary material for: Improved Environment-Aware–Based Noise Reduction System for Cochlear Implant Users Based on a Knowledge Transfer Approach: Development and Usability Study
Source: J Med Internet Res. 2021 Oct 28;23(10):e25460. doi: 10.2196/25460 (PMC8587190; doi:10.2196/25460)
Supplement: Multimedia Appendix 2 [file jmir_v23i10e25460_app2.docx]

## Appendix 2. Confusion Matrix of the 12 Noise Classifications

This appendix shows the confusion matrix of the of the noise classification model. The horizontal axis is the true class, and the vertical axis is the predicted class of the model. In other words, the accuracy, shown in the right bottom, is 100%. Therefore, the precision, recall, and F1 score are all equal to 100% which means all input data were judged correctly in this study.

**Figure A2.** Confusion Matrix of the 12 Noise Classifications.
