# Supplementary material for: Improved Environment-Aware–Based Noise Reduction System for Cochlear Implant Users Based on a Knowledge Transfer Approach: Development and Usability Study
Source: J Med Internet Res. 2021 Oct 28;23(10):e25460. doi: 10.2196/25460 (PMC8587190; doi:10.2196/25460)
Supplement: Multimedia Appendix 3 [file jmir_v23i10e25460_app3.docx]

## Appendix 4. Individual biographical data of the attended CI subjects

This section reveals the details of the subjects that attended the listening test. All of them were Taiwan Mandarin-speaking CI users. The average age of the 10 subjects is 41.3 and the standard deviation is 13.32. Noted that we only recruited CI users who had received their CI at least 7 months. The deafness of all the subjects were owing to sensorineural hearing loss (SNHL). Detailed individual biographical information for the 10 subjects is presented in Table A5.

| **Table A5.** Individual biographical data of the attended CI subjects. | | | | | | | |
| --- | --- | --- | --- | --- | --- | --- | --- |
| Subjects | Age (Years) | | Sex | Age at HL | CI Use (Years) | Etiology of Deafness | Processing Strategy |
| S1 | 53 | F | | child^*^ | 6 | SNHL | ACE |
| S2 | 57 | F | | 1 | 2 | SNHL | HiRes-P w/Fidelity 120 |
| S3 | 21 | F | | child^*^ | 1 | SNHL | HiRes Optima-P |
| S4 | 27 | M | | child^*^ | 6 | SNHL | HiRes-S w/Fidelity 120 |
| S5 | 27 | M | | 1 | 8 | SNHL | ACE |
| S6 | 49 | M | | 45 | 9 | SNHL | ACE |
| S7 | 31 | M | | 6 | 5 | SNHL | ACE |
| S8 | 58 | F | | 17 | 1 | SNHL | HiRes Optima-S |
| S9 | 38 | M | | 7 | 6 | SNHL | HiRes-P w/Fidelity 120 |
| S10 | 52 | M | | child^*^ | 1 | SNHL | HiRes Optima-P |

Because the subject couldn’t specify the actual age , child^*^ is used to include the age under 6 years old.
