# Supplementary material for: Improved Environment-Aware–Based Noise Reduction System for Cochlear Implant Users Based on a Knowledge Transfer Approach: Development and Usability Study
Source: J Med Internet Res. 2021 Oct 28;23(10):e25460. doi: 10.2196/25460 (PMC8587190; doi:10.2196/25460)
Supplement: Multimedia Appendix 4 [file jmir_v23i10e25460_app4.docx]

## Appendix 3. PESQ and STOI Scores of Different Noise Reduction Systems

This section shows the performances of different noise reduction systems in perceptual evaluation of speech quality (PESQ) and short-time objective intelligibility (STOI) scores corresponding to Figures 4 and 5. Each noise signal was mixed with the TMHINT corpus at 5signal-to-noise ratio levels. All corpus data were processed with noisy, deep denoising autoencoder (DDAE), noise classifier and DDAE (NC+DDAE), and NC+DDAE with knowledge transfer (NC+DDAE_T) approaches.

| **Table A1.** Perceptual evaluation of speech quality (PESQ) scores of each noise reduction system. | | | | | | |
| --- | --- | --- | --- | --- | --- | --- |
| Noise type |  | −6 dB | −3 dB | 0 dB | 3 dB | 6 dB |
| 2T_BG_1 | Noisy | 1.17645 | 1.28977 | 1.44407 | 1.6221 | 1.83749 |
|  | DDAE | 1.54590 | 1.70957 | 1.89785 | 2.06336 | 2.2564 |
|  | NC+DDAE | **2.09415** | **2.23715** | **2.39822** | **2.49706** | 2.61049 |
|  | NC+DDAE_T | 2.04021 | 2.20953 | 2.37429 | 2.49191 | **2.61650** |
| 2T_BG_2 | Noisy | 1.23333 | 1.33744 | 1.50055 | 1.69281 | 1.87679 |
|  | DDAE | 1.64385 | 1.82328 | 1.97234 | 2.16376 | 2.34730 |
|  | NC+DDAE | **2.05608** | **2.22143** | **2.37359** | 2.50763 | 2.61329 |
|  | NC+DDAE_T | 2.00445 | 2.18846 | 2.35237 | **2.51763** | **2.64091** |
| CJ | Noisy | 1.44297 | 1.56269 | 1.66890 | 1.81144 | 1.97955 |
|  | DDAE | 1.40357 | 1.63591 | 1.81817 | 1.99993 | 1.94071 |
|  | NC+DDAE | **1.91722** | **2.15437** | **2.30723** | **2.45712** | 2.60721 |
|  | NC+DDAE_T | 1.88731 | 2.11390 | 2.29116 | 2.45419 | **2.60821** |
| 2T_BB | Noisy | 1.34660 | 1.43930 | 1.61110 | 1.77667 | 1.95025 |
|  | DDAE | 1.41097 | 1.63433 | 1.81040 | 2.01435 | 1.93359 |
|  | NC+DDAE | **1.85888** | **2.04816** | **2.22860** | **2.37645** | **2.50192** |
|  | NC+DDAE_T | 1.82756 | 1.99451 | 2.16505 | 2.32402 | 2.4618 |

| MRT | Noisy | 1.25312 | 1.30110 | 1.38033 | 1.49112 | 1.60865 |
| --- | --- | --- | --- | --- | --- | --- |
|  | DDAE | 1.33613 | 1.45853 | 1.64239 | 1.82885 | 1.98643 |
|  | NC+DDAE | **1.6185** | **1.88500** | **2.10514** | **2.29142** | **2.44477** |
|  | NC+DDAE_T | 1.56823 | 1.85193 | 2.07191 | 2.25727 | 2.43719 |
| Cafeteria | Noisy | 1.36965 | 1.43716 | 1.56876 | 1.66656 | 1.81437 |
|  | DDAE | **1.55509** | 1.67045 | 1.79125 | 1.99326 | 2.15665 |
|  | NC+DDAE | 1.52993 | **1.79046** | **2.04642** | **2.26108** | 2.43833 |
|  | NC+DDAE_T | 1.48630 | 1.75854 | 2.03395 | 2.24431 | **2.44498** |
| House-Fan | Noisy | 1.21803 | 1.31979 | 1.46199 | 1.60337 | 1.77602 |
|  | DDAE | 1.42895 | 1.63949 | 1.83454 | 2.01738 | 2.17738 |
|  | NC+DDAE | **1.70628** | **1.94287** | 2.12669 | 2.31448 | 2.48934 |
|  | NC+DDAE_T | 1.67674 | 1.92679 | **2.14520** | **2.34080** | **2.52155** |
| Toy | Noisy | 1.21042 | 1.32365 | 1.42872 | 1.54899 | 1.70251 |
|  | DDAE | 1.73883 | 1.95724 | 2.11069 | 2.24469 | 2.12558 |
|  | NC+DDAE | **2.59462** | **2.70265** | **2.79900** | **2.89953** | **2.97429** |
|  | NC+DDAE_T | 2.49402 | 2.60439 | 2.70511 | 2.79698 | 2.89269 |
| SSN_IEEE | Noisy | 1.46906 | 1.56321 | 1.67623 | 1.81685 | 1.99071 |
|  | DDAE | 0.78179 | 0.98516 | 1.23768 | 1.54983 | 1.87583 |
|  | NC+DDAE | 1.38644 | 1.65659 | 1.95292 | 2.20568 | 2.42474 |
|  | NC+DDAE_T | **1.41179** | **1.71474** | **1.99672** | **2.25580** | **2.47390** |
| Siren | Noisy | 1.86316 | 1.89715 | 1.97068 | 2.06137 | 2.11730 |
|  | DDAE | 2.34045 | 2.47661 | 2.6084 | 2.71743 | 2.81766 |
|  | NC+DDAE | **2.69882** | 2.77961 | 2.86706 | 2.92439 | 2.97755 |
|  | NC+DDAE_T | 2.68787 | **2.78010** | **2.86935** | **2.96171** | **3.03783** |

| Multiple type noise 1 | Noisy | 1.68886 | 1.74786 | 1.80851 | 1.89012 | 1.99867 |
| --- | --- | --- | --- | --- | --- | --- |
|  | DDAE | 1.66164 | 1.88023 | 2.08009 | 2.26287 | 2.43388 |
|  | NC+DDAE | 1.89318 | 2.08293 | 2.24738 | 2.39328 | 2.51766 |
|  | NC+DDAE_T | **1.92933** | **2.13866** | **2.32350** | **2.48244** | **2.6223** |
| Multiple type noise 2 | Noisy | 1.30232 | 1.37532 | 1.47478 | 1.56544 | 1.72033 |
|  | DDAE | 1.53570 | 1.60805 | 1.74318 | 1.88297 | 2.08528 |
|  | NC+DDAE | **1.85450** | **2.03691** | **2.21555** | **2.35497** | **2.49586** |
|  | NC+DDAE_T | 1.73647 | 1.95311 | 2.15381 | 2.32559 | 2.49223 |

| **Table A2.** Short-time objective intelligibility (STOI) scores of each noise reduction system. | | | | | | |
| --- | --- | --- | --- | --- | --- | --- |
| Noise type |  | −6 dB | −3 dB | 0 dB | 3 dB | 6 dB |
| 2T_BG_1 | Noisy | 0.49281 | 0.56583 | 0.64080 | 0.70795 | 0.77668 |
|  | DDAE | 0.57272 | 0.62905 | 0.68268 | 0.72506 | 0.76258 |
|  | NC+DDAE | **0.70348** | 0.73416 | 0.76150 | 0.78121 | 0.79641 |
|  | NC+DDAE_T | 0.70317 | **0.73766** | **0.76525** | **0.78708** | **0.80343** |
| 2T_BG_2 | Noisy | 0.52073 | 0.60040 | 0.67323 | 0.74305 | 0.80517 |
|  | DDAE | 0.61422 | 0.66757 | 0.71058 | 0.74819 | 0.77739 |
|  | NC+DDAE | **0.71432** | **0.74700** | **0.77236** | **0.79171** | **0.80689** |
|  | NC+DDAE_T | 0.70212 | 0.73931 | 0.76617 | 0.78940 | 0.80662 |
| Construction Jackhammer | Noisy | 0.52978 | 0.60112 | 0.66755 | 0.73237 | 0.79219 |
|  | DDAE | 0.58017 | 0.64095 | 0.6918 | 0.73455 | 0.75065 |
|  | NC+DDAE | **0.68171** | **0.72536** | 0.75494 | 0.77922 | 0.79585 |
|  | NC+DDAE_T | 0.68169 | 0.72632 | **0.75809** | **0.78434** | **0.80394** |

| 2T_BB | | Noisy | | 0.50424 | | 0.59047 | | 0.66606 | | 0.74107 | | 0.80581 | |
| --- | --- | --- | --- | --- | --- | --- | --- | --- | --- | --- | --- | --- | --- |
|  |  | DDAE | | 0.58899 | | 0.63315 | | 0.68871 | | 0.73273 | | 0.74472 | |
|  |  | NC+DDAE | | **0.69696** | | **0.72840** | | **0.75566** | | **0.77782** | | 0.79607 | |
|  |  | NC+DDAE_T | | 0.68397 | | 0.72053 | | 0.74728 | | 0.77458 | | **0.79637** | |
| MRT | | Noisy | | 0.60664 | | 0.66323 | | 0.72138 | | 0.77481 | | 0.82301 | |
|  |  | DDAE | | 0.55351 | | 0.61367 | | 0.66791 | | 0.71822 | | 0.75343 | |
|  |  | NC+DDAE | | **0.68612** | | **0.73146** | | **0.76558** | | 0.**78906** | | **0.80760** | |
|  |  | NC+DDAE_T | | 0.67017 | | 0.7186 | | 0.75492 | | 0.78151 | | 0.80298 | |
| Cafeteria | | Noisy | | 0.54324 | | 0.61413 | | 0.68219 | | 0.74857 | | 0.80966 | |
|  |  | DDAE | | 0.72832 | | 0.75573 | | 0.77505 | | 0.78927 | | 0.78611 | |
|  |  | NC+DDAE | | **0.81233** | | **0.82293** | | **0.83153** | | 0.83742 | | 0.84098 | |
|  |  | NC+DDAE_T | | 0.80080 | | 0.81241 | | 0.82168 | | **0.82870** | | **0.83504** | |
| House-Fan | | Noisy | | 0.58486 | | 0.647 | | 0.71100 | | 0.77350 | | **0.82980** | |
|  |  | DDAE | | 0.56747 | | 0.63315 | | 0.68868 | | 0.73384 | | 0.76918 | |
|  |  | NC+DDAE | | **0.66120** | | **0.70760** | | 0.74327 | | 0.77604 | | 0.79966 | |
|  |  | NC+DDAE_T | | 0.65702 | | 0.70742 | | **0.74788** | | **0.78003** | | 0.80444 | |
| Toy | | Noisy | | 0.63013 | | 0.65837 | | 0.69407 | | 0.72791 | | 0.76234 | |
|  |  | DDAE | | 0.72832 | | 0.75573 | | 0.77505 | | 0.78927 | | 0.78611 | |
|  |  | NC+DDAE | | **0.81233** | | **0.82293** | | **0.83153** | | **0.83742** | | **0.84098** | |
|  |  | NC+DDAE_T | | 0.80080 | | 0.81241 | | 0.82168 | | 0.82870 | | 0.83504 | |
| SSN_IEEE | | Noisy | | 0.56720 | | 0.63893 | | 0.70986 | | **0.77977** | | **0.83828** | |
|  |  | DDAE | | 0.45235 | | 0.53648 | | 0.61874 | | 0.68877 | | 0.74781 | |
|  |  | NC+DDAE | | **0.59722** | | **0.65536** | | **0.70874** | | 0.75123 | | 0.78255 | |
|  |  | NC+DDAE_T | | 0.58422 | | 0.65132 | | 0.70818 | | 0.75948 | | 0.79360 | |

| Siren | Noisy | 0.65897 | 0.69741 | 0.74325 | 0.78434 | 0.82215 |
| --- | --- | --- | --- | --- | --- | --- |
|  | DDAE | 0.78078 | 0.79750 | 0.81327 | 0.82245 | 0.83075 |
|  | NC+DDAE | 0.81578 | 0.82347 | 0.82920 | 0.83327 | 0.83686 |
|  | NC+DDAE_T | **0.81880** | **0.82728** | **0.83356** | **0.83940** | **0.84302** |
| Multiple type noise 1 | Noisy | 0.59215 | 0.65726 | 0.71493 | 0.77270 | **0.82177** |
|  | DDAE | 0.63666 | 0.69599 | 0.73807 | 0.77391 | 0.79751 |
|  | NC+DDAE | 0.69485 | 0.73326 | 0.75953 | 0.78153 | 0.79612 |
|  | NC+DDAE_T | **0.70613** | **0.74561** | **0.77331** | **0.79639** | 0.81233 |
| Multiple type noise 2 | Noisy | 0.57848 | 0.63489 | 0.69550 | 0.75096 | 0.80404 |
|  | DDAE | 0.56100 | 0.61873 | 0.67400 | 0.72321 | 0.76344 |
|  | NC+DDAE | **0.71417** | **0.74656** | **0.77245** | **0.79203** | 0.80733 |
|  | NC+DDAE_T | 0.69558 | 0.73256 | 0.76349 | 0.78654 | **0.80563** |
